# The Contribution of the Underlying Factors to Socioeconomic Inequalities in Obesity: A Life Course Perspective

**DOI:** 10.3389/ijph.2024.1606378

**Published:** 2024-02-15

**Authors:** Yusong Dang, Xinyu Duan, Yaling Zhao, Jing Zhou, Lu Ye, Duolao Wang, Leilei Pei

**Affiliations:** ^1^ Department of Epidemiology and Health Statistics, Xi’an Jiaotong University, Xi’an, China; ^2^ The Second Affiliated Hospital of Xi’an Jiaotong University, Xi’an, China; ^3^ Xi’an No. 4 Hospital, Xi’an, China; ^4^ Department of Clinical Sciences, Liverpool School of Tropical Medicine, Liverpool, United Kingdom; ^5^ Key Laboratory of Age-Related Cardiac and Cerebral Diseases, Affiliated Hospital of Guangdong Medical University, Zhanjiang, China

**Keywords:** concentration index, obesity, socioeconomic inequality, Oaxaca decomposition, childhood and adulthood

## Abstract

**Objectives:** Socioeconomic disparities in obesity have been observed in both childhood and adulthood. However, it remains unclear how the role of risk factors influencing these inequalities has evolved over time.

**Methods:** Longitudinal data on 2,866 children and adolescents (6–17 years old) from the China Health and Nutrition Survey were used to track their BMI during childhood, adolescence, and adulthood. Concentration Index was utilized to measure socioeconomic inequalities in obesity, while Oaxaca decomposition was employed to determine the share of different determinants of inequality.

**Results:** The concentration index for obesity during childhood and adulthood were 0.107 (95% CI: 0.023, 0.211) and 0.279 (95% CI: 0.203, 0.355), respectively. Changes in baseline BMI (24.6%), parental BMI (10.4%) and socioeconomic factors (6.7%) were found to be largely responsible for the increasing inequality in obesity between childhood and adulthood. Additionally, mother’s education (−7.4%) was found to contribute the most to reducing these inequalities.

**Conclusion:** Inequalities in obesity during childhood and adulthood are significant and growing. Interventions targeting individuals with higher BMI, especially those who are wealthy, can significantly reduce the gap.

## Introduction

Obesity is a significant public health challenge of the 21st century. China, as a rapidly growing economy, has also experienced a quadrupling of obesity rates since 1975, and by 2014, it had the largest number of obese individuals [1]. Notably, among school-age children and adolescents aged above 7 in China, the prevalence of obesity has increased from 0.5% to 7.3% from 1985 to 2014 [2]. Childhood obesity is associated with metabolic health outcomes in adolescents, such as insulin resistance and high total cholesterol [3]. Given the high likelihood of obesity in children and adolescents persisting into adulthood [4, 5], a thorough examination of the developmental characteristics of obesity throughout the life-course is crucial for achieving significant reductions in the obesity-related health burden in adulthood.

Previous studies have demonstrated the effects of socioeconomic factors, diet and nutrition, and physical activity on obesity in adults and adolescents [6]. In particular, socioeconomic factors play an important role in the development of obesity in childhood and adulthood. A study in China indicated that the prevalence of pediatric obesity progressively increased with economic development, as indicated by Engel’s coefficients and urbanization levels [7]. A prospective 14-year follow-up study in China showed the pro-rich inequality in the prevalence of adult obesity [8]. To explore the reasons for inequality of adult obesity using the concentration index decomposition method, Zhou et al. indicated that urban residents, a college education or above were main contributing factors for pro-rich inequality of adult obesity [8]. To date, however, no studies have investigated the changes in obesity inequality throughout the life course or explored the underlying factors contributing to these changes.

In this study, we used a more than 20 years follow-up cohort to assess the inequality in obesity from school-age childhood to adulthood. Two main purposes of the study were 1) to evaluate the socioeconomic inequality of obesity in different life periods, including school-age childhood and adulthood; 2) to estimate the contribution of the underlying factors to socioeconomic inequality of obesity in the two different periods, and explore the reasons for the changing inequality of obesity in the follow-up periods.

## Methods

### Data and Participants

Data were derived from the China Health and Nutrition Survey (CHNS), which was an international collaborative project conducted by the Carolina Population Center at the University of North Carolina at Chapel Hill and the National Institute for Nutrition and Health at the Chinese Center for Disease Control and Prevention (CDC). The CHNS was first conducted in 1989, and subsequent surveys were performed in 1991, 1993, 1997, 2000, 2004, 2006, 2009, 2011 and 2015. A more detailed description of the design and procedures of CHNS has been described elsewhere [9].

The flow diagram of the study cohort is summarized in Supplementary Figure S1. A total of 9,212 participants aged 6 to 17 with 18,795 visits were extracted from the continuous longitudinal surveys during 1991–2011. However, 6,346 participants were excluded from the current analysis, including 2,667 participants with less than 2 visits of BMI in school-age childhood and adolescence, 3,666 participants with less than 2 visits of BMI in adulthood during 2004–2015, and 13 pregnant women. Finally, a total of 2,866 school-age children with 11,674 visits, were included in the final analytic sample.

### Outcome Variables

Both school-age children and adult obesity, as measured by BMI, were the main outcome variable in this study. BMI was calculated as weight (in kilograms) divided by the square of height (in meters). The obesity cutoffs use the China national standards, which are lower than the World Health Organization’s standards due to differences in body composition, genetics, and cultural factors in different populations [10]. More details about differences between the obesity category cutoffs could be seen in the Supplementary Text. According to the Working Group on Obesity in China (WGOC) [11], adult overweight and obesity are defined as 24 ≤ BMI < 28 kg/m^2^ and BMI ≥ 28 kg/m^2^, respectively. According to the China national standards (WS/T 586–2018), the overweight and obesity of school-age children aged 6–17 are age- and sex-specific [12]. Details about the BMI cutoffs were provided in Supplementary Table S1.

### Covariates

Based on previous studies and *a priori* knowledge about our data [13], we included several potential influencing factors for obesity in both childhood and adulthood, such as demographic characteristics, socioeconomic factors, and health behaviors. Demographic characteristics included sex (male or female), residence (urban or rural), and age of participants. For our study, age was categorized into three groups during the two different periods: 6–7 years, 8–9 years, and 10 years and older during childhood, and 18–19 years, 20–21 years, and 22 years and older during adulthood. For households with BMI measures for two parents, the classification of parental BMI was based on whether either person was overweight or obese. If both parents were not overweight or obese, they were classified as normal/underweight. If either person was obese, they were classified as obese, otherwise classified as overweight. Socioeconomic factors included parental education and household wealth index. Parental education was classified into three groups: low (<8 years), middle (8–11 years), and high (≥12 years).

Two independent household wealth indices were constructed through the principal component analysis for assessing economic status of participants in school-age childhood and adulthood respectively. The first principal component, which summarized the largest amount of information on an inventory of household assets or facilities, was used as the household wealth index [14, 15]. The HWI for school-age children and adolescents combined information on household ownership of durable goods (e.g., bicycle, private car, motorcycle, television, tape recorder, refrigerator, and washing machine), dwelling characteristics (type of dwelling, type of toilet facilities, materials used for house/apartment roof and floor), the major source of drinking water, and the type of lighting. Similarly, the HWI for adulthood synthesized information on ownership of computer and telephone, in addition to a set of household assets and living conditions mentioned above. Finally, the two HWI were divided into five quintiles: the 1st quintile represented the poorest households and 5th quintile the richest households.

Health behaviors in the study included sugary beverages consumption (SBC), the total dietary energy intake (TDEI), physical activity (PA), and sedentary behaviors. Sugary beverages consumption was measured by asking respondents if they drank soft/sugared fruit beverages in the last month (“No” and “Yes”). The dietary information at individual and household level, were collected through three consecutive-24 h recalls at two weekdays and one weekend. Total dietary energy intake was calculated by the 3-day average of total energy intake using the China Food Composition Table [16].

Adult physical activity (PA) included occupational, domestic, travel, and leisure physical activities. Among children and adolescent, PA included the sports in and outside school, travel, and domestic activities. The types and duration of physical activities were reported in average hours-per-week spent in the past year. The metabolic equivalent (MET, unit kcal/kg/h) was used to assess the intensity of the activities based on the Compendium of Physical Activities [17]. The PA was measured by the product of time and specific MET values of each activity. Finally, PA was categorized into three subgroups, representing light (percentile 0–25), moderate (percentile 25–75), heavy (over percentile 75) levels of PA among participants. The Sedentary behavior time were assessed using the average time per week (h/week) spent in various non-occupational recreational activities, such as watching TV, video/computer games, reading, surfing internet, watching videos/movies online, and others. The total time of sedentary behaviors was calculated by summing the duration of all the recreational activities. Respondents were asked to report leisure time hours only and to exclude time spent on these activities at work or school.

### Statistical Analysis

The baseline characteristics were presented as numbers (percentages) and mean ± SD between school-age childhood and adulthood. All data management and statistical analyses were conducted in STATA 12/SE and R version 4.1.3, and statistical significance was set at a 2-sided *p* < 0.05.

### Inequality Measurement

The concentration index (CI) proposed by Wagstaff and Van Doorslaer [18], accurately reflects the health inequalities originated from the socioeconomic factors. The concentration index is twice the area enclosed by the concentration curve and the line of equality (the 45-degree line). The CI ranges from −1 to +1, with the higher absolute value representing the stronger inequality of obesity [19]. The formula of CI is expressed as follows:

$$CI=\frac{2}{\mu }cov\left(h,r_{i}\right)$$ (1)

Where *r* is the cumulative proportion of individual *i* sorted by HWI, *h* is the health variable (e.g., adult and child obesity) and *μ* represents the average of the health variables. As obesity is a binary variable, based on previous experience [20, 21], the CI was normalized by means of the following formula:

$${CI}_{normalized}=\frac{CI}{1-\mu }$$ (2)

The Stata Conindex command is used to estimate the concentration index and its standard error.

#### Decomposition of Inequality Index

To explore the possible reasons for socioeconomic inequalities in obesity, the CI decomposition approach proposed by Wagstaff was adopted [22]. The contribution of each underlying factor to inequality in obesity equals to the product of the elasticity and the concentration index of each factor. Since the outcome variables in this study are binary categorical variables, the marginal effects on the logit model were used to approximate the decomposition analysis [23]:

$$y_{i}=\alpha^{m}+\sum_{k}\beta_{k}^{m}x_{ki\;}+\varepsilon_{i}$$ (3)

Where $\beta_{k}^{m}$ is the marginal effect (dy/dx) of independent variables; $\varepsilon_{i}$ signifies the error term generated by the linear approximation. The decomposition of the concentration index is expressed as follows:

$$CI=\sum \left(\frac{\beta_{k}^{m}{\bar{x}}_{k}}{\mu }\right)C_{k}+\frac{GC_{\varepsilon }}{\mu }$$ (4)

In Formula 4, ${\bar{x}}_{k}$ and *µ* is the mean of independent and dependent variables, respectively. $\left(\frac{\beta_{k}^{m}{\bar{x}}_{k}}{\mu }\right)$ is the elasticity of dependent variable with respect to $x_{k}$ , $C_{k}$ is the CI of independent variables, and ( $\frac{GC_{\varepsilon }}{\mu }$ ) is the error term. $\frac{\beta_{k}^{m}{\bar{x}}_{k}}{\mu }C_{k}$ represents the contributions of independent variables, with the positive value indicating that the certain factor favors the better-off and increased the probability of being obese among the rich and *vice versa* [19]. Applying Wagstaff’s correction into Formula 4 results in:

$${CI}_{normalized}=\frac{CI}{1-\mu }=\frac{\sum \left(\frac{\beta_{k}^{m}{\bar{x}}_{k}}{\mu }\right)C_{k}}{1-\mu }+\frac{{GC}_{\varepsilon }/\mu }{1-\mu }$$ (5)

### Oaxaca-Type Decomposition of CI Change Over Time

The Oaxaca-type decomposition method was used to measure the contributions of the underlying factors to the changing inequality in obesity from school-age childhood to adulthood [24]. According to the method, the changes in SES-related inequality in obesity were attributed to changes in inequality in the determinants of health, and changes in the elasticities of obesity with respect to these determinants. The positive value of change in each variable represents that the variable increases the total change of obesity inequality and *vice versa*.

$$△CI=\sum_{k}\eta_{kt}\left(C_{kt}-C_{kt-1}\right)+\sum_{k}C_{kt-1}\left(\eta_{kt}-\eta_{kt-1}\right)+△\left(\frac{GC_{\varepsilon t}}{\mu_{t}}\right)$$ (6)

where *k* represents the total number of independent variables, *t* refers to the survey time and Δ denotes the first differences. η is elasticity of CI, calculated by $\left(\frac{\beta_{k}^{m}{\bar{x}}_{k}}{\mu }\right)$ .

### Sensitivity Analysis

To ensure the robustness of our results, the concentration index and Oaxaca decomposition were recalculated by using *per capita* household income to reduce the error of different measures of household economic status. However, due to the missing data on parental and offspring household income, the analysis sample would be further reduced to 2,155. Next, we utilized multiple imputation by chained equations models to handle missing covariates. As the maximum amount of missing data for these variables was approximately 5%, we generated 20 imputed datasets. The decomposition of the concentration index was repeated using each of the 20 amplified datasets, and the parameter estimates were averaged across all 20 sets. We compared the contribution of each factor before and after multiple imputation to assess the impact of missing data on our findings. Multiple imputation using chained equations was also used with missing household income data to test the reliability of obesity inequality as measured by household income.

## Results

In the study, a total of 2,866 participants were included, of which 59.9% (*N* = 1717) were male (Table 1). Specifically, 71.50% of participants lived in rural areas during their school-age childhood and adolescence, and 65.80% of them continued to live in rural areas in adulthood. The levels of sugary beverage consumption, moderate and heavy physical activity, total dietary energy intake, and sedentary behavior time were much lower in the school-age childhood, compared to the adulthood. At the follow-up endpoint, the prevalence of obesity for school-age children was 5.4% (the International Obesity Task Force criteria [25]: 2.5%). And the prevalence of adult obesity was 8.2% (WHO criteria [26]: 4.2%), which was similar to the finding of adult obesity reported in the 2002 CHNS data (China criteria: 7.1%, WHO criteria: 2.9%) [27]. A logistic regression model was fitted to explore the influencing factors associated with obesity in school-age C&A and adulthood. The results were presented in the Supplementary Table S2, and showed that most determinants of obesity between C&A and adulthood were similar except for sex, mother’s education and SBC.

**TABLE 1:** Baseline demographic characteristics of participants according to different life periods in China 1991–2015.

Variables	Categories	School-age C&A		Adulthood	
		*N*	Percent (%)	*N*	Percent (%)
Sex^a^	Male	1,717	59.90		
	Female	1,149	40.10		
Father’s education^a^	<8 years	458	16.00		
	8–11 years	1,876	65.50		
	≥12 years	532	18.60		
Mother’s education^a^	<8 years	656	22.90		
	8–11 years	1,655	57.70		
	≥12 years	555	19.40		
Parental BMI^a^	Normal/Underweight	585	20.40	585	20.40
	Overweight	1,415	49.4	1,415	49.4
	Obesity	866	30.2	866	30.2
Residence	Rural	2,050	71.50	1,883	65.80
	Urban	816	28.50	983	34.20
Age^b^	6–7	1,153	40.20	1,921	67.00
	8–9	790	27.60	456	15.90
	≥10	923	32.20	489	17.10
BMI	Normal/Underweight	2,552	89.00	2,416	84.30
	Overweight	169	5.90	312	10.90
	Obesity	145	5.10	138	4.80
HWI	Poorest quintile	589	20.60	570	19.90
	2nd quintile	558	19.50	576	20.10
	3rd quintile	574	20.00	572	20.00
	4th quintile	571	19.90	572	20.00
	Richest quintile	574	20.00	576	20.10
SBC	No	2,305	80.40	1,856	64.80
	Yes	556	19.40	1,006	35.10
PA	Light PA	853	29.80	436	15.20
	Moderate PA	1,776	62.00	1,814	63.30
	Heavy PA	226	7.90	399	13.90
TDEI (kcal/day)		1964.76 ± 785.93		2,270.50 ± 729.79	
SBT (h/week)		1.63 ± 3.69		7.31 ± 8.55	

C&A, childhood and adolescence; HWI, household wealth index; SBC, sugary beverage consumption; SBT, sedentary behavior time; PA, physical activity; TDEI, The total dietary energy intake.

^a^ This implied that these variables were categorized with the same frequency between the two periods.

^b^ Age in adulthood is also tri-categorized: 18–19, 20–21, and ≥22 years of age.

**FIGURE 1:**
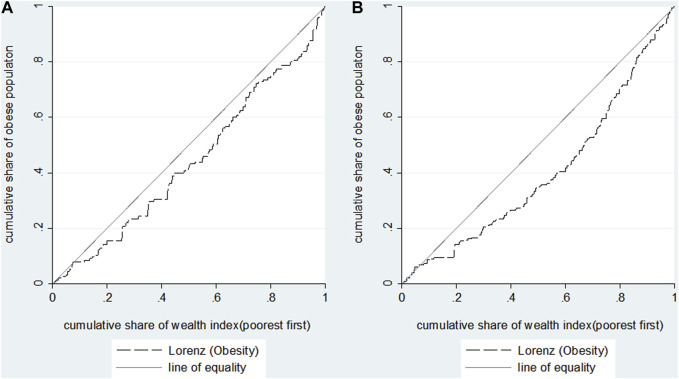
Concentration curves for obesity during childhood and adolescence **(A)** and adulthood **(B)** in China 1991–2015.

In Table 2, the results showed that the concentration indices of obesity increased from 0.107 (95% CI: 0.023, 0.211) in C&A to 0.279 (95% CI: 0.203, 0.355) with a growth rate of 160.74%. The positive concentration indices suggested that participants with higher HWI were more likely to suffer from obesity both in childhood and adulthood. Similarly, Figure 1 indicated that concentration curves of obesity were below the line of equality. The analysis of adult obesity inequalities across various sexes and areas of the residence revealed that obesity was primarily prevalent among affluent individuals. Nonetheless, the concentration indices failed to demonstrate statistical significance (*p* > 0.1) in the school-age children and adolescents, regardless of sex or place of residence.

**TABLE 2:** Concentration indices for obesity during different life periods, China 1991–2015.

	Concentration index	
	School-age C&A	Adulthood
Sex		
Male	0.101 (−0.021, 0.223)	0.283*** (0.194, 0.371)
Female	0.113 (−0.031, 0.256)	0.274*** (0.117, 0.430)
Residence		
rural	0.083 (−0.035, 0.201)	0.330*** (0.235, 0.425)
urban	0.072 (−0.081, 0.224)	0.138** (0.009, 0.267)
Total	0.107** (0.023, 0.211)	0.279*** (0.203, 0.355)
CI_adu_- CI_ado_	0.172	

The numbers in () indicate a 95% confidence interval; C&A, childhood and adolescence; CI, concentration index; _ado_, adolescence; _adu_, adulthood.

***p < 0.01, ** *p* < 0.05, *p < 0.1.

Further, we conducted the CI decomposition analysis to identify how much a certain factor contributed to the inequalities in obesity. In C&A and adulthood, the elasticity, and CIs of each factor, and the absolute contribution and relative contribution percentages of each factor to the total CI, were all reported in Table 3. It was observed that high age, high parental education, high HWI and long sedentary behavior time were concentrated among the rich, whereas rural residents, and high TDEI were more concentrated among the poor in school-age childhood and adolescence. In adulthood, the distributions of age, parental education, HWI, SBC, SBT were consistent with that in childhood, while heavy PA and TDEI were more likely to occur among the poor.

**TABLE 3:** Decomposition of inequalities in obesity during different life periods in China 1991–2015.

	School-age C&A					Adulthood				
	E	CI	Contri.	Percentage contri. (%)	Summed percentage contri. (%)	E	CI	Contri.	Percentage contri. (%)	Summed percentage contri. (%)
Sex										
Male										
Female	0.249	0.011	0.003	2.45	2.45	−0.480	−0.004	0.002	0.67	0.67
Residence										
Rural										
Urban	−0.086	0.250	−0.021	−20.00	−20.00	−0.045	0.112	−0.005	−1.81	−1.81
Age^a^										
6–7 years										
8–9 years	−0.122	0.023	−0.003	−2.60		0.010	0.068	0.001	0.25	
≥10 years	−0.189	0.107	−0.020	−18.74	−21.35	−0.005	0.445	−0.002	−0.84	−0.60
BMI										
Normal/Underweight										
Overweight	0.051	0.152	0.008	7.29		0.059	0.160	0.009	3.37	
Obesity	0.123	0.046	0.006	5.21	12.50	0.131	0.353	0.046	16.59	19.96
Parental BMI										
Normal/Underweight										
Overweight	0.056	−0.001	0.000	−0.04		0.124	0.030	0.004	1.34	
Obesity	0.045	0.048	0.002	2.03	1.99	0.114	0.143	0.016	5.83	7.17
HWI										
Poorest quintile										
2nd quintile	−0.002	−0.485	0.001	1.06		−0.005	−0.500	0.002	0.82	
3rd quintile	0.035	0.033	0.001	1.08		0.038	0.000	0.000	−0.01	
4th quintile	0.029	0.504	0.015	13.61		0.045	0.505	0.023	8.19	
Richest quintile	0.059	1.000	0.059	55.35	71.10	0.063	1.000	0.063	22.40	31.40
Mother’s education										
<8 years										
8–11 years	0.135	−0.086	−0.012	−10.85		0.023	0.116	0.003	0.96	
≥12 years	0.047	0.369	0.017	16.13	5.28	−0.026	0.368	−0.010	−3.49	−2.53
Father’s education										
< 8 years										
8–11 years	−0.017	−0.068	0.001	1.06		0.122	0.085	0.010	3.70	
≥12 years	0.063	0.323	0.020	18.97	20.03	0.080	0.300	0.024	8.62	12.33
SBC										
Not										
Yes	−0.014	0.086	−0.001	−1.14	−1.14	0.022	0.179	0.004	1.41	1.41
PA										
Light PA										
Moderate PA	−0.042	−0.033	0.001	1.32		−0.070	−0.007	0.001	0.19	
Heavy PA	−0.076	0.029	−0.002	−2.06	−0.74	0.005	−0.209	−0.001	−0.37	−0.18
TDEI (kcal/day)	0.137	−0.002	0.000	−0.25	−0.25	0.121	−0.048	−0.006	−2.08	−2.08
SBT (h/week)	0.056	0.047	0.003	2.49	2.49	0.103	0.163	0.017	6.04	6.04

C&A, childhood and adolescence; E, elasticity; CI, concentration index; Contri., contribution; HWI, household wealth index; SBC, sugary beverage consumption; PA, physical activity; TDEI, the total dietary energy intake; SBT, Sedentary behavior time.

^a^ Age in adulthood is also tri-categorized: 18–19, 20–21, and ≥22 years of age.

**FIGURE 2:**
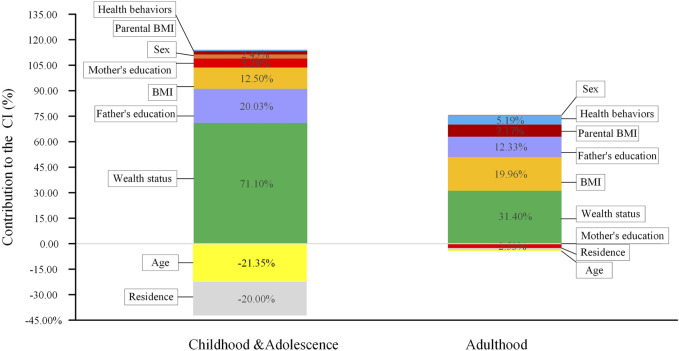
Decomposition of the concentration index for obesity, childhood and adolescence (C&A) and adulthood, China 1991–2015.

We assessed the contribution of each factor to the CI of obesity, based on the proportion of the relationship between obesity and HWI explained by the variation in a given explanatory factor. In school-age C&A, the HWI made the greatest contribution (71.10%) to the overall pro-rich inequality of obesity. Other factors in childhood such as high parental education (25.31%), baseline BMI (12.50%), SBT (2.49%) and sex (2.45%) were also important determinants that contributed to the observed pro-rich inequality in obesity. On the contrary, urban residents (−20.00%), higher age (−21.35%) increased the probability of being obese among the poor. In adulthood, the results showed that the mother’s education, urban area, higher age had a negative contribution to CI of obesity. It was found that HWI had the largest contribution to obesity inequality in C&A and adulthood (71.10% and 31.40%, respectively). The changing contribution of the underlying factors to obesity CI were presented in Figure 2.

According to the Oaxaca-type decomposition method, the total changes in inequalities from school-age childhood to adulthood were presented in Table 4. The 7th column showed changes in the magnitude of inequality with respect to the contributors and the 6th column showed changes in the elasticity of the obesity in these contributors. From childhood to adulthood, the observed characteristics accounted for 70.39% of the change in obesity inequality, of which 22.33% explained by demographic characteristics, 6.75% by socioeconomic factors, and 7.18% by health behaviors. While the remaining 29.61% was due to unobserved characteristics (residual term). Baseline BMI was the main contributor, contributing to 24.62% of the change in inequalities. Furthermore, approximately 14.16% of the increase in obesity inequalities was attributed to HWI and father’s education. However, sex (−0.45%), the total dietary energy intake (−3.90%), and mother’s education contributed (−7.41%) lessened the inequalities from childhood to adulthood.

**TABLE 4:** Oaxaca-type decomposition of change in inequalities for obesity in China 1991–2015.

	School-age C&A		Adulthood		(E_adu_ - E_ado_)	(CI_adu_-CI_ado_)	ΔCI	ΔE	Total	
	E_ado_	CI_ado_	E_adu_	CI_adu_					Change	(%)
Sex										
Male										
Female	0.249	0.011	−0.480	−0.004	−0.729	−0.014	0.007	−0.008	−0.001	−0.45
Residence										
Rural										
Urban	−0.086	0.250	−0.045	0.112	0.041	−0.138	0.006	0.010	0.016	9.54
Age^a^										
6–7 years										
8–9 years	−0.122	0.023	0.010	0.068	0.132	0.045	0.000	0.003	0.003	
≥10 years	−0.189	0.107	−0.005	0.445	0.183	0.339	−0.002	0.020	0.018	12.34
Baseline BMI										
Normal/Underweight										
Overweight	0.051	0.152	0.059	0.160	0.007	0.008	0.000	0.001	0.002	
Obesity	0.123	0.046	0.131	0.353	0.009	0.307	0.040	0.000	0.041	24.62
Parental BMI										
Normal/Underweight										
Overweight	0.056	−0.001	0.124	0.030	0.068	0.031	0.004	0.000	0.004	
Obesity	0.045	0.048	0.114	0.143	0.069	0.095	0.011	0.003	0.014	10.41
HWI										
Poorest quintile										
2nd quintile	−0.002	−0.485	−0.005	−0.500	−0.002	−0.015	0.000	0.001	0.001	
3rd quintile	0.035	0.033	0.038	0.000	0.003	−0.034	−0.001	0.000	−0.001	
4th quintile	0.029	0.504	0.045	0.505	0.016	0.001	0.000	0.008	0.008	
Richest quintile	0.059	1.000	0.063	1.000	0.003	0.000	0.000	0.003	0.003	6.64
Mother’s education										
<8 years										
8–11 years	0.135	−0.086	0.023	0.116	−0.112	0.203	0.005	0.010	0.014	
≥12 years	0.047	0.369	−0.026	0.368	−0.073	0.000	0.000	−0.027	−0.027	−7.41
Father’s education										
<8 years										
8–11 years	−0.017	−0.068	0.122	0.085	0.138	0.153	0.019	−0.009	0.009	
≥12 years	0.063	0.323	0.080	0.300	0.017	−0.023	−0.002	0.006	0.004	7.52
SBC										
Not										
Yes	−0.014	0.086	0.022	0.179	0.036	0.092	0.002	0.003	0.005	2.91
PA										
Light PA										
Moderate PA	−0.042	−0.033	−0.070	−0.007	−0.028	0.026	−0.002	0.001	−0.001	
Heavy PA	−0.076	0.029	0.005	−0.209	0.081	−0.238	−0.001	0.002	0.001	0.17
TDEI (kcal/day)	0.137	−0.002	0.121	−0.048	−0.015	−0.046	−0.006	0.000	−0.006	−3.90
SBT (h/week)	0.056	0.047	0.103	0.163	0.047	0.116	0.012	0.002	0.014	8.00
Totals									0.121	70.39
Residual									0.051	29.61
Difference (C_adu_ - C_ado_)									0.172	

C&A, childhood and adolescence; E, elasticity; CI, concentration index; _ado_, adolescence; _adu_, adulthood; HWI, household wealth index; SBC, sugary beverage consumption; PA, physical activity; TDEI, The total dietary energy intake; SBT, Sedentary behavior time.

^a^ Age in adulthood is also tri-categorized: 18–19, 20–21, and ≥22 years of age.

The results of concentration index calculated by the *per capita* household income showed that the concentration indices of obesity in C&A and adulthood were 0.106 (0.022, 0.190) and 0.267 (0.179, 0.355), respectively (Supplementary Figure S2; Supplementary Table S3). The results of Oaxaca decomposition remained consistent with the main findings (Supplementary Table S4). In addition, a sensitivity analysis showed that the trends remained unchanged in the multiple imputation analysis (Supplementary Figure S3). Details about missing covariates were shown in the Supplementary Table S5. After using multiple imputations to impute missing values of all covariates, we repeated all analyses and observed similar findings (Supplementary Figures S4, S5).

## Discussion

Our study findings indicated that individuals with higher household wealth index (HWI) were at a greater risk of obesity during both childhood and adulthood. Moreover, the concentration index (CI) for obesity was 1.6 times higher in adulthood than in childhood, indicating that obesity was more concentrated among affluent participants in adulthood compared to childhood. Previous studies in China also showed that children or adults with high socioeconomic status had a higher risk of obesity, and CIs of adult obesity ranged from 0.01 to 0.15 [28, 29]. And the pro-rich inequality continued to aggravate from childhood to adulthood in China [2, 8]. In mid-low income countries like Iran, it was reported that CIs of obesity in childhood were between 0.03 and 0.10 [30, 31]. In the Europe and US, however, the socioeconomic status was often found to be inversely associated with child and adolescent obesity [32–34], and the similar relationship was observed among adult women, except for adult men [35, 36]. Raftopoulou and Trasfi combined CI and Foster-Greer-Thorbecke index to estimate the inequality in obesity beyond the obesity threshold and found that inequalities in depth and severity of obesity were much greater for the poor compared to the rich in Spain [37]. In the developing countries, the rich may have easier access to sufficient calories, which could lead to the positive relationship between SES and obesity. With economic growth and introduction of the western lifestyle in the developing countries, diets full of fat and sugar also tend to be cheaper and consumed by people with lower SES [38]. In the developing countries, obesity seems to repeat the historical experience of the developed regions, where sedentary behaviors are popular, and people with higher SES may engage in more leisure physical activity or healthy lifestyle [39], which explained the opposite socioeconomic gradients for obesity in the developing countries.

According to the CI decomposition analysis, the study confirmed that the baseline BMI and socioeconomic factors as indicated by the household wealth index and the parental education, were the major contributors to the pro-rich-inequality of obesity in school-age childhood and adulthood. These findings are consistent with previous research indicating that socioeconomic factors play a critical role in overall inequality. For example, a study in western Iran showed that socioeconomic factors accounted for 75.8% of the inequalities in childhood obesity [40]. The other study among Chinese workers found individual wage played a key role for pro-rich obesity both in males (77.68%) and females (41.56%) [28]. The results from a Spanish study indicated that household income contributed to 65.9% of the equality in childhood obesity [41]. It is widely acknowledged that household socioeconomic status influences the physical environment in which individuals are exposed. Food intake and lifestyles at different life stages are fundamentally influenced by socioeconomic status [42]. Economic factors contributed the most to obesity inequality in the decomposition analysis based on either *per capita* household income or the household wealth index, this reminded us to enhance dietary and exercise-related health education for the newly wealthy.

Our study found that high levels of father’s education were associated with increased obesity risk in both childhood and adulthood, while maternal education had a similar impact on childhood obesity but was associated with reduced obesity inequality in adulthood. Previous research in mid-low income countries has also confirmed that childhood obesity is more prevalent among children from wealthier or better-educated families [43]. In China, paternal education is often used as a proxy for the family’s socioeconomic status and can have a significant impact on child development [44]. Some earlier studies have shown that the relationship between education and obesity shifts from positive to negative with social development [45]. As a country undergoes a nutritional transition, education may help prevent an increased risk of overweight or obesity. These findings may explain the opposite contribution of maternal education to obesity inequality from childhood to adulthood observed in our study.

According to the Oaxaca-type decomposition, BMI at baseline had the greatest impact on the total change in obesity inequalities, accounting for 24.62% of the total change. This finding is consistent with the research of Simmonds et al., which demonstrated that obese children and adolescents are more likely to stay obese in adulthood than those who are not obese [4]. Therefore, the detection and control of BMI in childhood are crucial for reducing obesity in adulthood. The parental BMI contributed to 10.41% of the increase in obesity inequalities across the two periods. The culture of cooking and eating habits learned from parents can be transmitted to children, and parental obesity might be transmitted to their offspring through this [41].Urban residence contributed to 9.54% of the increase in obesity inequalities. With economic transition and urbanization, the western lifestyle has become more popular in urban areas, increasing the risk of obesity [43]. Our results showed that HWI and father’s education contributed to the increase in obesity inequalities over time. Higher levels of father’s education, means a greater increase in family income and more access to material resources [46]. In contrast, mother’s education accounted for a substantial decrease in obesity inequalities, suggesting a protective effect of maternal education on individual obesity over time. This highlights the critical role of maternal education in reducing inequalities in the health burden imposed by obesity. However, the changes in obesity inequalities over time were also influenced by other unobserved factors, with a residual of 29.61%. Therefore, further research is needed to incorporate additional variables to better explain obesity inequality.

Our study has several notable strengths. First, a long-term cohort enabled us to obtain complete data and analyze obesity inequality trends over time. Second, the Oaxaca-type decomposition allowed us to fill certain knowledge gaps in long-term obesity inequality trends among Chinese participants. Despite the strengths of our study, several limitations should be noted. First, the information on lifestyle behaviors was self-reported by participants at baseline, and the possibility of information bias cannot be entirely ruled out. Physical activity was derived from a questionnaire, which may lead to recall bias. However, the questionnaire, which was derived from the internationally used PA questionnaire, was widely accepted by the participants and was the most cost-effective and useful method for conducting a large-scale survey in nine provinces in China. To control the measurement error in our study, a multivariate general linear model was used to estimate objective measurements of each physical activity through self-reported data. For each model, the independent variable was the self-reported PA, with the covariates age, sex, and the level of education [47]. Second, although CHNS is a nationally representative sample, only 2,866 eligible adults were included. The reason for this was that in our analyses less than 50% of the individuals could match the parent-child relationship. In addition, tracking the socioeconomic status of two generations requiring a longer time span, will lead to more missing data. However, the results of sensitivity analysis considering the *per capita* household income, showed that the associations were robust. Third, although the lower BMI thresholds have been widely adopted in China, the obesity rates estimated using these standards are higher than those estimated using the WHO standards, which may lead to different results to be further explored.

### Conclusion

This study confirms the existence of pro-rich inequality in obesity in China, with an increasing trend from childhood to adulthood. Baseline BMI, urban residents, older age, higher HWI, higher father’s education and parental BMI were associated with an increase in inequality of obesity from childhood to adulthood, while high mother’s education and female sex were associated with a decrease in inequality. By targeting high-risk groups, such as the wealthy with higher BMI, a comprehensive approach to obesity prevention and control should take into account both individual and environmental factors.

## Data Availability

China Health and Nutrition Survey data are available in a public, open access website (https://www.cpc.unc.edu/projects/china/data/datasets). The data that support the findings of this study are available from the corresponding author.

## Abbreviations

C&A, Childhood and adolescence; CHNS, China Health and Nutrition Survey; HWI, household wealth index; SBC, Sugary beverage consumption; SES, socioeconomic status; SBT, Sedentary behavior time; PA, Physical activity; TDEI, The total dietary energy intake; E, Elasticity; CI, Concentration index; Contri, Contribution.
