# Supplementary material for: The Contribution of the Underlying Factors to Socioeconomic Inequalities in Obesity: A Life Course Perspective
Source: Int J Public Health. 2024 Feb 15;69:1606378. doi: 10.3389/ijph.2024.1606378 (PMC10902784; doi:10.3389/ijph.2024.1606378)
Supplement: Supplementary file 1 [file DataSheet1.PDF]

Additional file of contents:

Additional file 1: Text.

Additional file 1: Table S1. Body mass index (BMI) thresholds for overweight and obesity in Chinese school-age children and adolescents aged 6-18, by sex and age group

Additional file 1：Table S2. Multivariate regression model for determinants of obesity, school-age C&A and adulthood

Additional file 1：Table S3. Concentration indices for obesity during school-age C&A and adulthood (measured by per capita household income)

Additional file 1：Table S4. Oaxaca-type decomposition of change in inequalities for obesity, C&A and adulthood (measured by per capita household income).

Additional file 1：Table S5. Details about missing covariates

Additional file 1: Figure S1. Flow diagram for cohort selection and censure

Additional file 1：Figure S2. Concentration curves for obesity during childhood and adolescence (A) and adulthood (B), measured by per capita household income

Additional file 1：Figure S3. Sensitivity analysis of change in the decomposition of the concentration index for obesity (before & after Multiple Imputation) measured by the per capita household income

Additional file 1：Figure S4. Sensitivity analysis of decomposition of the concentration index for obesity (before & after Multiple Imputation)

Additional file 1：Figure S5. Sensitivity analysis of change in the decomposition of the concentration index for obesity (before & after Multiple Imputation)

Additional file 1: Text

The obesity category cutoffs differ between the China national standards and the World Health Organization (WHO) standards.

General obesity in adults

WHO[1]

BMI of 25.0-29.9 kg/m² for overweight and ≥30.0 kg/m² for obesity

Working Group on Obesity in China[2]

BMI of 24.0–27.9 kg/m² for overweight and ≥28.0 kg/m² for obesity

Obesity in children and adolescents

International Obesity Task Force[3]

For children and adolescents aged 2-18 years, BMI greater than sex-specific and age-specific cutoffs based on the assumptions of passing 25.0 (for overweight) and 30.0 kg/m² (for obesity) at age 18 years.

China Obesity Task Force[4]

For children and adolescents aged 7-18 years, BMI greater than cutoffs corresponding to the 85th (for overweight) and 95th (for obesity) centile of sex-specific and age-specific cutoffs based on the assumptions of passing 24.0 (for overweight) and 28.0 kg/m² (for obesity) at age 18 years.

The rationale behind these differences stems from several factors. Firstly, China has a higher prevalence of obesity and related health issues compared to the global average. Therefore, the lower cutoffs in China reflect the need to address the specific health concerns associated with obesity within the Chinese population. Secondly, there are variations in body composition and genetic factors among different populations. The China national standards consider the unique characteristics of the Chinese population when defining obesity categories. Lastly, cultural and societal factors also play a role. China traditionally values lean physiques, and there is a perception that higher BMI levels may be linked to increased health risks. Thus, the lower cutoffs are in line with the cultural emphasis on maintaining a healthy weight and preventing obesity-related health problems. The lower BMI cutoffs have been widely used in China, the studies consistently support the use of lower BMI cutoffs in Chinese than those in whites[5,6].

---

1 Zhou B-F, on behalf of the Cooperative Meta-Analysis Group of the Working Group on Obesity in China. Predictive values of body mass index and waist circumference for risk factors of certain related diseases in Chinese adults—study on optimal cut-off points of body mass index and waist circumference in Chinese adults. Biomed Environ Sci 2002; 15: 83–96.

2 Puska P, Nishida C, Porter D, World Health Organization. Obesity and overweight. World Health Organization 2003:1–2.

3 Cole TJ, Bellizzi MC, Flegal KM, Dietz WH. Establishing a standard definition for child overweight and obesity worldwide: international survey. BMJ 2000;320:1240.

4 National Health and Family Planning Commission of the People's Republic of China. WS/T 586–2018 Screening for overweight and obesity among school-age children and adolescents. 2018.

5 World Health Organization. Regional Office for the Western Pacific.(2000).The Asia-Pacific perspective : redefining obesity and its treatment. Sydney: Health Communications Australia.

6 He W, Li Q, Yang M, Jiao J, Ma X, Zhou Y, Song A, Heymsfield SB, Zhang S, Zhu S. Lower BMI cutoffs to define overweight and obesity in China. Obesity (Silver Spring). 2015 Mar;23(3):684-91.

Table S1. Body mass index (BMI) thresholds for overweight and obesity in Chinese school-age children and adolescents aged 6-18, by sex and age group [a]

Age (Year)	Male		Female	
	Overweight	Obesity	Overweight	Obesity
6.0~	16.4	17.7	16.2	17.5
6.5~	16.7	18.1	16.5	18.0
7.0~	17.0	18.7	16.8	18.5
7.5~	17.4	19.2	17.2	19.0
8.0~	17.8	19.7	17.6	19.4
8.5~	18.1	20.3	18.1	19.9
9.0~	18.5	20.8	18.5	20.4
9.5~	18.9	21.4	19.0	21.0
10.0~	19.2	21.9	19.5	21.5
10.5~	19.6	22.5	20.0	22.1
11.0~	19.9	23.0	20.5	22.7
11.5~	20.3	23.6	21.1	23.3
12.0~	20.7	24.1	21.5	23.9
12.5~	21.0	24.7	21.9	24.5
13.0~	21.4	25.2	22.2	25.0
13.5~	21.9	25.7	22.6	25.6
14.0~	22.3	26.1	22.8	25.9
14.5~	22.6	26.4	23.0	26.3
15.0~	22.9	26.6	23.2	26.6
15.5~	23.1	26.9	23.4	26.9
16.0~	23.3	27.1	23.6	27.1
16.5~	23.5	27.4	23.7	27.4
17.0~	23.7	27.6	23.8	27.6
17.5~	23.8	27.8	23.9	27.8
18.0~	24.0	28.0	24.0	28.0

[a] National Health and Family Planning Commission of the People's Republic of China. (2018). WS/T 586–2018 Screening for overweight and obesity among school-age children and adolescents. (http://www.nhc.gov.cn/wjw/pqt/201803/a7962d1ac01647b9837110bfd2d69b26.shtml)

Table S2. Multivariate regression model for determinants of obesity, school-age C&A and adulthood

Variables	School-age C&A			Adulthood		
	Odds Ratio.	[95% Conf. Interval]		Odds Ratio.	[95% Conf. Interval]	
Sex (Ref. Male)						
Female	1.492**	1.106	2.459	0.342***	0.193	0.605
Residence (Ref. Urban residence)						
Rural	0.582*	0.323	0.947	0.935	0.402	1.453
Age† (Ref. 6-7 years)						
8-9 years	0.295***	0.148	0.587	0.897	0.504	1.599
≥10 years	0.159***	0.059	0.432	0.579*	0.295	0.959
BMI (Ref. Normal or underweight)						
Overweight	6.997***	3.653	13.403	2.901***	1.840	4.575
Obesity	231.244***	129.376	413.318	217.806***	106.918	443.699
Parental BMI (Ref. Normal or underweight)						
Overweight	1.336	0.626	2.851	1.641	0.867	3.107
Obesity	1.665*	1.073	2.585	2.109**	1.088	4.085
Wealth status (Ref. Poorest quintile)						
2nd quintile	1.062	0.451	2.5	1.155	0.36	1.343
3rd quintile	1.815	1.056	2.574	1.450**	1.193	3.507
4th quintile	1.223	0.531	2.816	1.555	0.732	3.304
Richest quintile	1.815**	1.276	4.813	1.868**	1.159	3.719
Mother's education (Ref. Below 8 years)						
8-11 years	1.506	0.818	2.775	0.441***	0.238	0.816
≥12 years	2.481**	1.554	3.408	0.659	0.289	1.504
Father's education (Ref. Below 8 years)						
8-11 years	1.54	0.833	2.845	0.910	0.543	1.523
≥12 years	3.300***	1.564	6.964	2.350**	1.062	5.200
SBC (Ref. Not)						
Yes	0.837	0.420	1.669	1.113	0.755	1.697
PA (Ref. Light PA)						
Moderate PA	0.738	0.398	1.368	0.816*	0.399	0.935
Heavy PA	0.232*	0.024	0.926	0.996	0.491	2.058
TDEI (kcal/day)	1.075**	1.002	1.148	1.225**	1.008	1.485
SBT(h/week)	1.057*	1.019	1.128	1.047***	1.027	1.069
Constant	0.008***	0.001	0.051	0.030***	0.006	0.160

*** *P*<0.01, ** *P*<0.05, * *P*<0.1

C&A: Childhood and adolescence; SBC: Sugary beverage consumption; PA: Physical activity; TDEI: The total dietary energy intake; SBT: Sedentary behavior time

† Age in adulthood is also tri-categorized: 18-19, 20-21, and ≥22 years of age；

Table S3. Concentration indices for obesity during school-age C&A and adulthood (measured by per capita household income)

	Concentration index	
	School-age C&A	Adulthood
Total	0.106** (0.022, 0.190)	0.267***(0.179, 0.355)
Change	0.161	

C&A: Childhood and adolescence; CI: Concentration index;
*** P<0.01, ** P<0.05, * P<0.1

Table S4. Oaxaca-type decomposition of change in inequalities for obesity, C&A and adulthood (measured by per capita household income).

	School-age C&A		Adulthood		$(E_{adu} - E_{ado})$	$(CI_{adu}-CI_{ado})$	ΔCI	ΔE	Total	
	E_{ado}	CI_{ado}	E_{adu}	CI_{adu}					Change	(%)
Gender										
Male										
Female	0.028	0.176	-0.063	-0.066	-0.091	-0.242	0.015	-0.016	-0.001	-0.41
Residence										
Rural										
Urban	-0.094	0.043	-0.069	0.025	0.024	-0.018	0.001	0.001	0.002	1.35
Age †										
6-7 years										
8-9 years	-0.031	-0.002	-0.007	-0.045	0.024	-0.043	0.000	0.000	0.000	
≥10 years	-0.132	0.079	0.002	0.379	0.134	0.300	0.001	0.011	0.011	6.65
Baseline BMI										
Normal/Underweight										
Overweight	0.042	0.067	0.079	0.137	0.037	0.070	0.006	0.002	0.008	
Obesity	0.095	0.117	0.135	0.255	0.040	0.138	0.019	0.005	0.023	18.19
Parental BMI										
Normal/Underweight										
Overweight	-0.023	-0.009	0.154	0.049	0.177	0.058	0.009	-0.002	0.007	

Obesity	-0.016	0.082	0.147	0.068	0.163	-0.014	-0.002	0.013	0.011	10.89
HWI										
Poorest quintile										
2nd quintile	0.009	-0.498	0.013	-0.500	0.004	-0.002	0.000	-0.002	-0.002	
3rd quintile	0.034	0.000	0.061	0.000	0.027	0.000	0.000	0.000	0.000	
4th quintile	0.019	0.500	0.070	0.500	0.051	0.000	0.000	0.025	0.025	
Richest quintile	0.034	1.000	0.059	1.000	0.025	0.000	0.000	0.025	0.025	28.19
Mother's education										
< 8 years										
8-11 years	0.186	0.057	0.043	0.045	-0.143	-0.011	0.000	-0.008	-0.009	
≥12 years	0.017	0.302	-0.012	0.335	-0.029	0.033	0.000	-0.009	-0.009	-10.32
Father's education										
< 8 years										
8-11 years	-0.084	0.040	0.157	0.011	0.242	-0.029	-0.005	0.010	0.005	
≥12 years	0.027	0.256	0.089	0.239	0.062	-0.017	-0.002	0.016	0.014	11.21
SBC										
Not										
Yes	-0.095	0.189	-0.039	0.196	0.056	0.007	0.000	0.011	0.010	6.03
PA										
Light PA										
Moderate PA	-0.006	-0.018	-0.105	0.038	-0.100	0.056	-0.006	0.002	-0.004	

Heavy PA	-0.092	0.077	0.004	-0.183	0.096	-0.260	-0.001	0.007	0.006	1.39
TDEI (kcal/day)	-0.022	-0.014	0.103	-0.021	0.125	-0.006	-0.001	-0.002	-0.002	-1.43
SBT(h/week)	0.077	0.136	0.122	0.177	0.046	0.041	0.005	0.006	0.011	6.53
Totals									0.135	83.71
Residual									0.026	16.29
Difference (C_{adu} - C_{ado})									0.161	

C&A: Childhood and adolescence; E: Elasticity; CI: Concentration index; $_{ado}$: adolescence; $_{adu}$: adulthood; HWI: household wealth index; SBC: Sugary beverage consumption; PA: Physical activity; TDEI: The total dietary energy intake； SBT: Sedentary behavior time；

† Age in adulthood is also tri-categorized: 18-19, 20-21, and ≥22 years of age;

Table S5. Details about missing covariates

Variables	Missing ratio (%)	
	School-age childhood	Adulthood
SBC	5(0.2)	4(0.1)
PA	11(0.4)	217(7.6)
TDEI	8(0.3)	214(7.4)

SBC: Sugary beverage consumption; PA: Physical activity; TDEI: The total dietary energy intake;

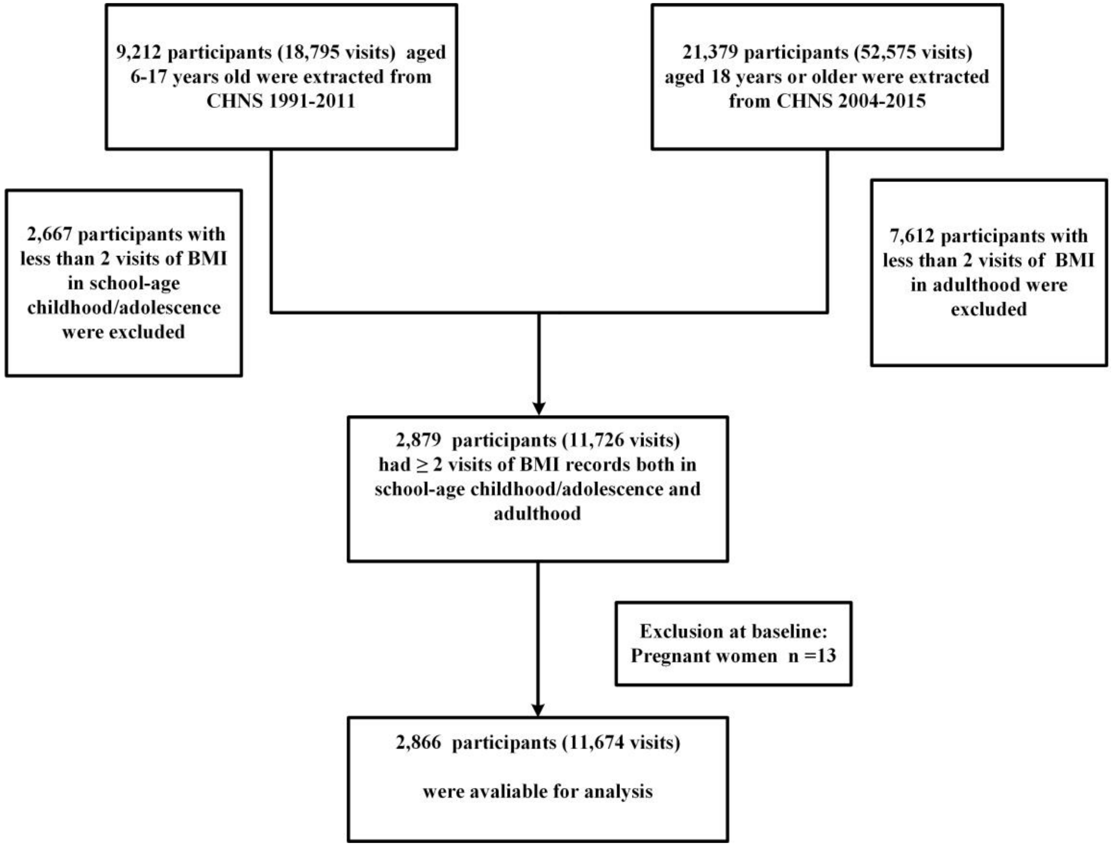

Figure S1. Flow diagram for cohort selection and censure

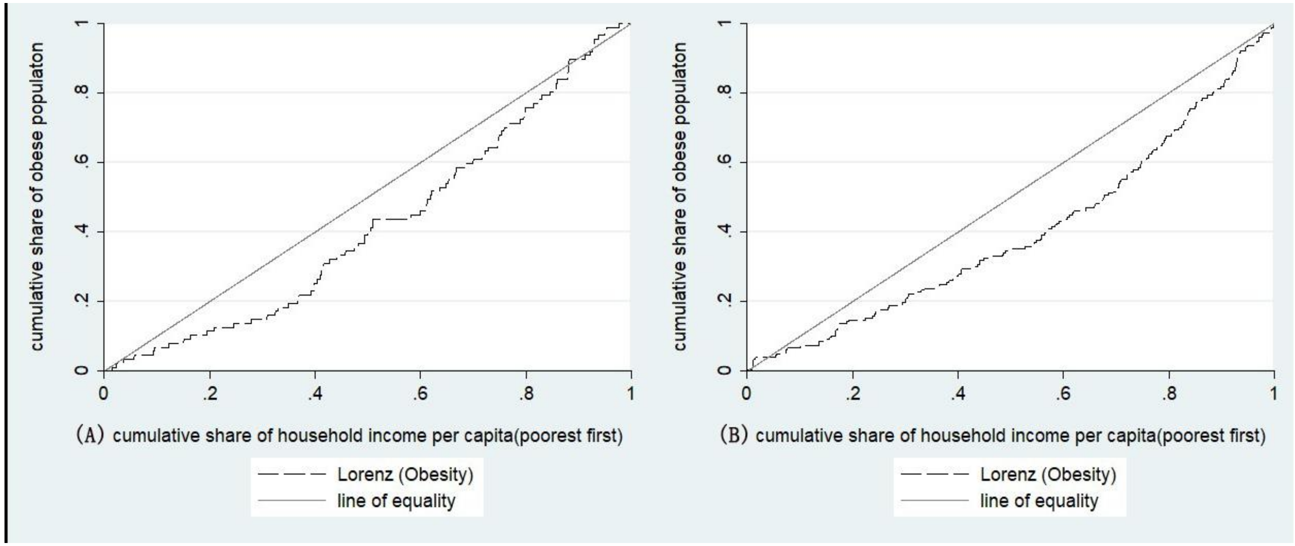

Figure. S2 Concentration curves for obesity during C&A (A) and adulthood (B), measured by per capita household income

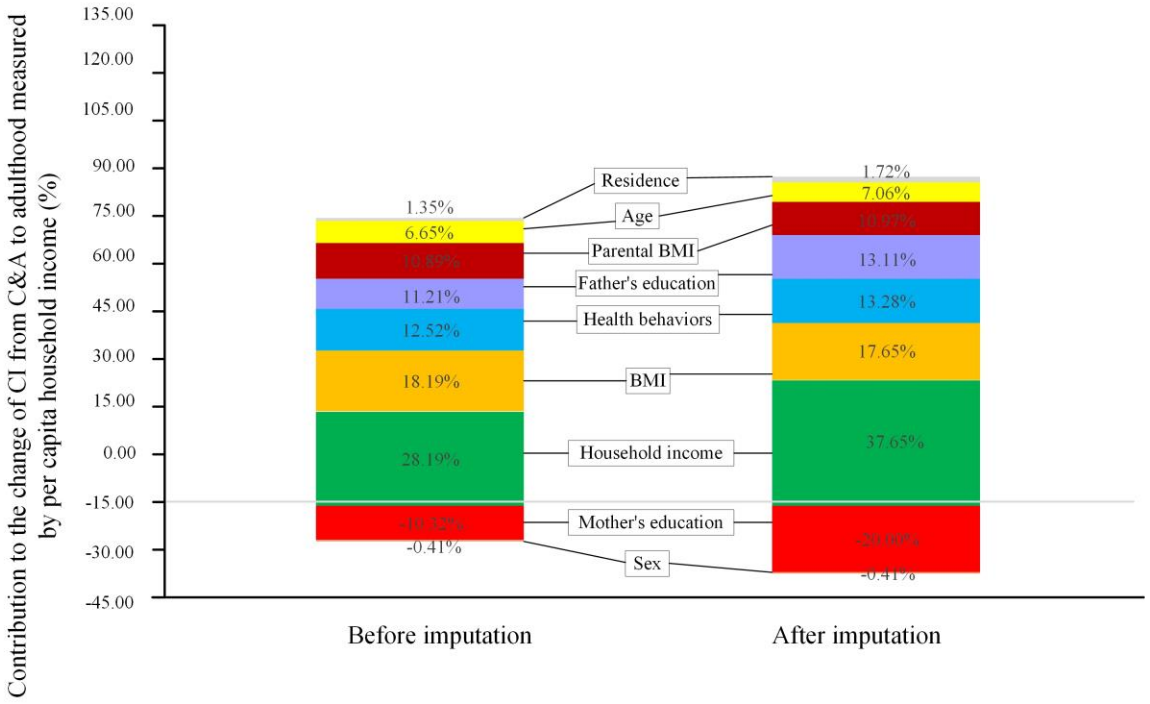

Figure S3. Sensitivity analysis of change in the decomposition of the concentration index for obesity (before & after Multiple Imputation) measured by the per capita household income

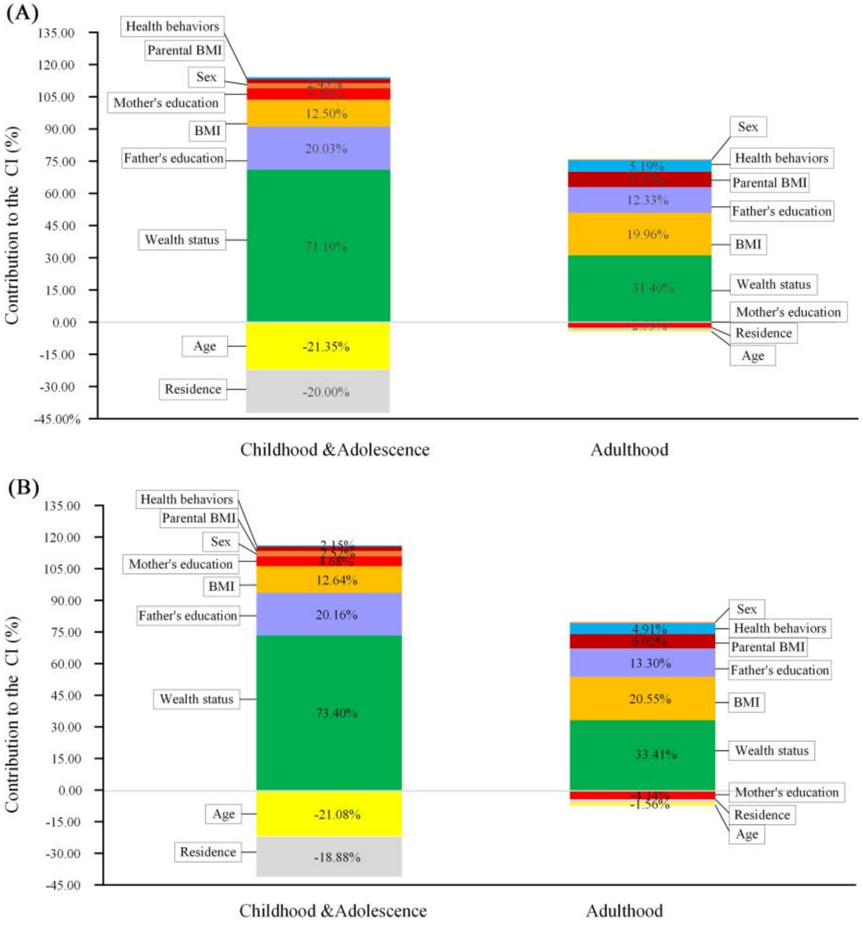

Figure S4. Sensitivity analysis of decomposition of the concentration index for obesity (before (A) & after Multiple Imputation (B))

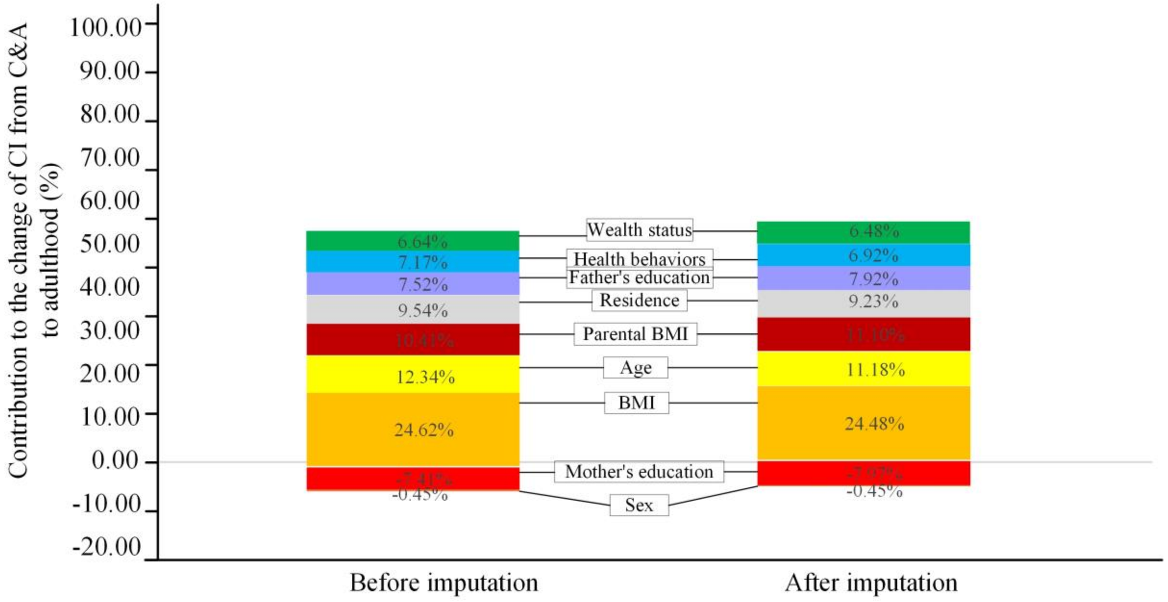

Figure S5. Sensitivity analysis of change in the decomposition of the concentration index for obesity (before & after Multiple Imputation)
